# Supplementary material for: Siglec receptors impact mammalian lifespan by modulating oxidative stress
Source: eLife. 2015 Apr 7;4:e06184. doi: 10.7554/eLife.06184 (PMC4384638; doi:10.7554/eLife.06184)
Supplement: Supplementary file 1. — Pathway analysis, calculated with Ingenuity Pathway Analysis software. The p-value is a measure of the likelihood genes in the process appear by chance and it was calculated by right-tailed fisher exact test. The z-score is calculated by IPA software algorithm, and predicts the change in the biological function direction. A z-score ≥ 2 or ≤ −2 is considered significant. DOI: http://dx.doi.org/10.7554/eLife.06184.024 [file elife06184s001.docx]

**Supplementary file 1. Pathway analysis, calculated with Ingenuity Pathway Analysis software.** The *p*-value is a measure of the likelihood genes in the process appear by chance and it was calculated by right-tailed fisher exact test. The z-score is calculated by IPA software algorithm, and predicts the change in the biological function direction. A z-score ≥ 2 or ≤ ‑2 is considered significant.

Metabolism of ROS *p*-value = 8.04x10-5, z-score = 2.030

| ID | Genes in dataset | Log Ratio |
| --- | --- | --- |
| Hsp90ab1 | HSP90AB1 | 1.470 |
| Hc | C5 | 1.698 |
| Hba-a1 | HBA1/HBA2 | -1.390 |
| Txnip | TXNIP | 1.501 |
| Itih4 | ITIH4 | 2.164 |
| Vcam1 | VCAM1 | 1.815 |
| Aifm1 | AIFM1 | 1.041 |
| Cyp2a5 | CYP2A6 (includes others) | 1.128 |
| Mapk14 | MAPK14 | 1.018 |
| C3 | C3 | 1.375 |
| S100a8 | S100A8 | 2.004 |
| Tgm2 | TGM2 | 1.395 |
| **Gstp1** | **GSTP1** | **-1.374** |
| Col18a1 | COL18A1 | 1.386 |
| Agt | AGT | 1.089 |
| Icam1 | ICAM1 | 1.017 |
| Msrb2 | MSRB2 | -1.454 |
| Cygb | CYGB | 1.040 |
| Snca | SNCA | -1.610 |
| Socs3 | SOCS3 | 1.052 |
| Hspa9 | HSPA9 | 1.067 |
| Dusp1 | DUSP1 | 1.231 |
| Serpinf1 | SERPINF1 | 1.203 |
| Spp1 | SPP1 | 1.051 |
| Prdx5 | PRDX5 | -1.675 |
| Dhcr24 | DHCR24 | 1.417 |
| F2r | F2R | 1.471 |
| Idh1 | IDH1 | 1.606 |
| Lgals3 | LGALS3 | 1.402 |
| Actb | ACTB | 1.312 |

Activation of leukocytes *p*-value = 7.3x10-8, z-score = 3.751

| ID | Genes in dataset | Log Ratio |
| --- | --- | --- |
| Sirpa | SIRPA | 1.404 |
| Hc | C5 | 1.698 |
| Hsph1 | HSPH1 | 1.685 |
| Txnip | TXNIP | 1.501 |
| Vtn | VTN | 1.250 |
| Cish | CISH | 1.551 |
| Vcam1 | VCAM1 | 1.815 |
| Hspd1 | HSPD1 | 1.500 |
| Lgals3 | LGALS3 | 1.402 |
| Dpp4 | DPP4 | 1.111 |
| Mapk14 | MAPK14 | 1.018 |
| C3 | C3 | 1.375 |
| S100a8 | S100A8 | 2.004 |
| Dusp1 | DUSP1 | 1.231 |
| Serpinf1 | SERPINF1 | 1.203 |
| Hsp90b1 | HSP90B1 | 1.970 |
| Lcn2 | LCN2 | 2.394 |
| Pros1 | PROS1 | 1.277 |
| Lbp | LBP | 1.120 |
| Spp1 | SPP1 | 1.051 |
| Chuk | CHUK | 1.178 |
| Vegfa | VEGFA | 1.473 |
| Hamp2 | Hamp/Hamp2 | 2.341 |
| Serpinf2 | SERPINF2 | 1.195 |
| Notch1 | NOTCH1 | 1.075 |
| Agt | AGT | 1.089 |
| Icam1 | ICAM1 | 1.017 |
| Plg | PLG | 1.434 |
| Snca | SNCA | -1.610 |
| Axl | AXL | 1.020 |
| Cd81 | CD81 | 1.036 |
| Vamp8 | VAMP8 | -1.097 |
| Serping1 | SERPING1 | 1.337 |
| Ppp3cb | PPP3CB | 1.087 |
| H2-Ab1 | HLA-DQB1 | 1.156 |
| Bhlhe40 | BHLHE40 | 1.208 |
| Ctss | CTSS | 1.248 |
| S100a9 | S100A9 | 1.216 |
| Parp1 | PARP1 | 1.322 |
| Tgm2 | TGM2 | 1.395 |
| Ahnak | AHNAK | 1.125 |
| Cd59b | CD59 | -1.207 |
| Ptpn11 | PTPN11 | 1.465 |
| C4b | C4A/C4B | 1.476 |
| Laptm5 | LAPTM5 | 1.713 |
| Adam9 | ADAM9 | 1.142 |
